# Genome Sequence of Oenococcus oeni OE37, an Autochthonous Strain Isolated from an Italian White Wine

**DOI:** 10.1128/MRA.00582-20

**Published:** 2020-09-24

**Authors:** Antonella Costantini, Ana B. Blazquez, Francesco Cerutti, Enrico Vaudano, Simone Peletto, Juan-Carlos Saiz, Emilia Garcia-Moruno

**Affiliations:** a Consiglio per la Ricerca in Agricoltura e l’Analisi dell’Economia Agraria, Research Centre for Viticulture and Enology, Asti, Italy; b Departamento de Biotecnología, Instituto Nacional de Investigación y Tecnología Agraria y Alimentaria, Madrid, Spain; c Istituto Zooprofilatico Sperimentale del Piemonte, Liguria e Valle d’Aosta, Torino, Italy; Portland State University

## Abstract

Oenococcus oeni OE37 is an autochthonous strain that was isolated from a Chardonnay wine from Piedmont (Italy) during spontaneous malolactic fermentation. Here, the OE37 genome sequence is presented, and a brief description of the main genes is reported.

## ANNOUNCEMENT

Malolactic fermentation (MLF) is a biological process in which malic acid is converted to lactic acid and carbon dioxide by decarboxylation through the NADH-malic enzyme (1). Oenococcus oeni is the main bacterium responsible for conducting this process because of its ability to survive the harsh wine conditions and its production of desirable wine sensory attributes. Currently, the most significant studies have been focused on describing the occurrence of MLF, lactic acid bacteria, and O. oeni starter selection (2, 3). The number of reported O. oeni genomes is increasing, but only 4 of 242 records currently available in GenBank are complete genome sequences.

OE37 is an autochthonous strain that was isolated from a Chardonnay wine during spontaneous MLF by plating diluted wine onto MRS agar plates, as described by Doria et al. (4). In this study, the OE37 strain was multiplied in MRS broth, and DNA was purified using the ArchivePure yeast/Gram-positive DNA kit (Eppendorf, Milan, Italy). Whole-genome sequencing was performed by Macrogen (South Korea). A library was prepared using the TruSeq Nano DNA kit, and sequencing was performed using the Illumina HiSeq 2500 platform, with a paired-end read length of 101 bp. Trimmomatic v0.36 (5) was used to remove adapter sequences; after trimming, a total of 23,084,810 reads, with 96.72% of bases having a quality score above Q30, were found. The GC content was 37.53%, and the mean coverage depth was 1,132.21×. Reads were mapped, using *Oenococcus* PSU-1 as the reference genome (GenBank accession number NC_008528.1), with BWA v0.7.17 (6) and SAMtools v1.9-1 (7). For all software, default parameters were used.

The sequence was annotated by the National Center for Biotechnology Information (NCBI) Prokaryotic Genome Annotation Pipeline (PGAP). The resulting draft complete sequence is 1,718,702 bp, with a GC content of 37.99%, and contains 1,720 coding sequences, 46 tRNAs, and 6 rRNA-like motifs. Assembly completeness was checked with Benchmarking Universal Single-Copy Orthologs (BUSCO) v.1 (8) on the gVolante server (9), using bacteria as the selected reference gene set, which resulted in 97.5% completeness.

In winemaking, different aspects are considered for starter selection, such as the ability to tolerate the harsh wine conditions and physiological, biochemical, and technological properties (10). The observation of the genes contained in the OE37 genome confirmed the ability of this bacterium to conduct MLF, since malic enzyme is present; genes related to citrate metabolism are also present, as is a gene coding for diacetyl reductase. Diacetyl is the main aromatic compound associated with MLF and is derived from citrate consumption (11). Concerning the organoleptic quality, other genes are involved and are present in this strain, such as various glucosidase enzyme genes, including that for a β-glucosidase (12, 13).

Regarding the mechanism of the stress response of O. oeni, several genes previously reported as being implicated in the response are present in the OE37 genome, such as those involved in cell wall biosynthesis (e.g., *N*-acetylmuramoyl-l-alanine amidase and d-alanyl-d-alanine carboxypeptidase), whose expression is strongly influenced by the presence of ethanol (14, 15). Genes coding for exopolysaccharides, which are important for the adaptation of O. oeni to its ecological niche (16), are also present.

The genome sequence of this O. oeni strain confirms the current knowledge regarding malolactic bacteria and their impact on wine.

### Data availability.

The genome sequence was deposited in GenBank under accession number CP053280. The raw sequencing data were deposited in the SRA database under accession number PRJNA607182.
